# Single mission workload and influencing factors in German prehospital emergency medicine - a nationwide prospective survey of 1361emergency missions

**DOI:** 10.1186/s13049-019-0650-2

**Published:** 2019-08-16

**Authors:** Johannes Prottengeier, Johann Georg Keunecke, Christine Gall, Christian Eiche, Andreas Moritz, Torsten Birkholz

**Affiliations:** 1 Department of Anaesthesiology, University Hospital Erlangen, Erlangen, Germany; 2 Faculty of Medicine, Friedrich-Alexander University Erlangen-Nuremberg, Erlangen, Germany; 3 Department of Medical Informatics Biometry and Epidemiology, Friedrich-Alexander University Erlangen-Nuremberg, Erlangen, Germany

**Keywords:** Emergency medical service, Paramedic, Workload, NASA task-load-index, Teamwork, Human factors, Ergonomics

## Abstract

**Background:**

Workload is a major determinant of system performance and human well-being. This study aims to evaluate workload in prehospital emergency medicine on a single mission level and investigates influencing factors originating from medical scenarios, patient-provider interaction, EMS logistics and teamwork.

**Methods:**

In a nationwide study, German paramedics were asked to evaluate single missions for perceived workload by completing the NASA Task-Load-Index (TLX). A variety of candidate variables were documented and tested for influence on the TLX through multivariate regression analysis.

**Results:**

One thousand three hundred sixty-one emergency missions were analysed. Global workload scored in medium ranges (Median TLX 41.00/100; IQR 24.25–57.50). 263 missions achieved very low (< 20/100) and 52 missions achieved very high (> 80/100) levels of workload. Severity of distress as indicated by the NACA score (delta TLX 2.71 per 1 NACA point), execution of invasive procedures (e.g. delta TLX 8.20 for intravenous access), obese patients (delta TLX 0.05 per 1 kg of weight) and aggression incidences (e.g. delta TLX 10.54 for physical aggression), amongst others, resulted in significant increases in workload. Good teamwork decreased workload by 2.18 points per 1 point on the Weller-Teamwork Measurement Tool.

**Conclusion:**

Distinct factors result in significant increases in workload for EMS paramedics. Improvements in training for certain medical scenarios, strategies against aggression events and enhancements in EMS logistics - especially for the transfer of obese patients – should be implemented and tested for their presumably positive effect on workload, EMS performance and paramedics’ well-being.

**Electronic supplementary material:**

The online version of this article (10.1186/s13049-019-0650-2) contains supplementary material, which is available to authorized users.

## Background

Emergency medical services (EMS) aim for fast and accurate performance by their trained professionals in a multitude of scenarios. In this, the human factor remains pivotal in patient care in spite of all pharmaceutical and technical advances. Only by achieving the highest levels in human performance will we be able to provide the best possible outcome for an emergency patient. The discipline of ergonomics and human factors can help us to understand the intricate interrelationship of human performance, required tasks and external conditions [1]. By understanding and optimizing ergonomics, we can increase system performance in EMS and increase well-being for paramedics.

One major factor that human performance is dependent on is “workload”. The term workload represents the proportion of one individual’s capacity that needs to be mobilized to perform a given task. Workload comprises, amongst others, physical and mental domains. It can be measured physiologically and/or subjectively for short-term single tasks as well as long-term assignments [2].

There exists an inversely U-shaped correlation between workload and human performance. A medium level of workload leads to optimal output, while extremes on both sides, i.e. excessively low and excessively high workload will lead to decreased performance [3]. This has also been demonstrated for a medical context where workload indices and treatment errors were positively correlated [4, 5].

In spite of the vital importance of ergonomics for human performance, there is only limited data available regarding workload in prehospital emergency medicine. Only recently has the feasibility of workload-assessment in emergency medicine been demonstrated for a cohort of emergency physicians [6]. For the larger group of EMS paramedics, such data is not available.

Our study aims to assess the single-mission workload of EMS paramedics during emergency runs by means of the NASA task-load-index (NASA-TLX) [7]. To examine possible influencing factors on work-load, we explored a variety of candidate variables, comprising, amongst others, medical and organizational characteristics of emergency missions as well as exchanges with patients and perceived teamwork between paramedics using the teamwork measurement tool (TMT) by Weller [8, 9].

We formed a collaboration network of EMS stakeholders to disseminate our investigation across Germany. The goal was to generate the largest cohort possible and thus provide representative data on a national level.

## Methods

The Friedrich-Alexander-University of Erlangen-Nuremberg’s research ethics committee approved of our study beforehand through formal decision number 172_17B. Our endeavour was endorsed by EMS providers (Bayerisches Rotes Kreuz, Arbeiter Samariter Bund Bayern) as well as the EMS paramedic association of Germany (Bundesverband Rettungsdienst) and the paramedics’ labour union (Ver.di Bayern – Landesfachbereich Gesundheit).

In the autumn of 2017, we conducted our nationwide prospective survey in Germany. The study was promoted extensively. Advertisements inviting paramedics to participate were placed in all major German EMS medical journals. Information letters, posters and leaflets were sent to all German EMS stations and paramedic academies.

Our survey was executed as an online questionnaire, available through the platform Sosci-Survey. Participation was voluntary, non-paid and anonymous.

All types of missions within the scope of the German EMS were eligible for subjective assessment and documentation. This paper reports on emergency missions only. Accordance with these criteria was required for mission documentation to be eligible for analysis: Less than 20% of the questionnaires’ items unanswered in total, complete TLX scoring, mission classified as a medical emergency. The findings from documented non-emergency patient transfers will be discussed separately elsewhere.

Workload was measured as a subjective self-assessment after each mission using the National Aeronautics and Space Administration’s Task-Load-Index (NASA-TLX) by Hart and Staveland [7]. The TLX is highly sensitive to workload alterations and robust against individual differences of workload perception. It is considered one of the most reliable tools for workload quantification and as such has found widespread use in human factors research. Hart and Staveland postulate that workload can be divided into six equally weighted subdimensions. Mental, physical and temporal demands, as well as performance, effort and frustration with respect to the task at hand, are rated on scales from 1 to 100. To get a better understanding of these subdimensions, we present the actual wording of each TLX-item in Table 1. Summation and averaging of the 6 sub-scale values results in a global workload score between 1 (low) and 100 (high).

**Table 1:** Wording of the NASA TLX questionnaire

TLX-dimension	Wording (scale)
Mental Demand	How much mental and perceptual activity was required? Was the task easy or demanding, simple or complex? Was high precision required or was the task fault-tolerant? (1 = low, 100 = high)
Physical Demand	How much physical activity was required? Was the task easy or demanding, slack or strenuous? (1 = low, 100 = high)
Temporal Demand	How much time pressure did you feel due to the pace at which the tasks or task elements occurred? Was the pace slow or rapid? (1 = low, 100 = high)
Overall Performance	How successful were you in reaching your goals or the goals set by your team leader? How satisfied were you with your performance? (1 = good, 100 = bad)
Effort	How hard did you have to work to accomplish your level of performance? (1 = low, 100 = high)
Frustration	How irritated, stressed, and annoyed versus content, relaxed, and complacent did you feel during the task? (1 = low, 100 = high)

The selection of candidate variables as possible influencing factors on workload was derived through discussion by an expert panel at the Erlangen University Hospital. As a core feature, the patients’ condition as classified by the National Advisory Committee for Aeronautics (NACA) score was obtained. (Table 2 shortly summarizes the NACA scoring.) In addition, we documented tracer diagnoses as they are defined by governmental EMS oversight in Germany. They are certain clinical scenarios like multiple trauma, cerebral insult and others that are deemed unique in their setting and consequences.

**Table 2:** Injury or disease severity scoring as originally described by the National Advisory Committee on Aeronautics (NACA)

NACA 1	Injuries/diseases without any need for acute physician care
NACA 2	Injuries/diseases requiring examination and therapy by a physician, but hospital admission is not indicated
NACA 3	Injuries/diseases without an acute threat to life but requiring hospital admission
NACA 4	Injuries/diseases that can possibly lead to deterioration of vital signs
NACA 5	Injuries/diseases with an acute threat to life
NACA 6	Injuries/diseases transported after successful resuscitation of vital signs
NACA 7	Lethal injuries or diseases

The list further comprised logistical factors of EMS missions, medical procedures performed, the patients’ weight and several others. Communication and feedback with patients, relatives and bystanders were also recorded.

A special focus was laid on team interactions. To assess teamwork amongst paramedics and dealings with emergency physicians we utilized the Teamwork Measurement Tool (TMT) by Weller [8, 9]. It is a self-assessment instrument of 23 items on 7-point Likert-scales to measure team behaviour and teamwork in three subdimensions: “Leadership and Team Coordination”, “Mutual Performance Monitoring” and “Verbalising Situational Information”. The TMT was specifically developed to assess teamwork in emergency situations and ratings can also be issued as values on a percentage scale.

Data analysis was conducted in SPSS Statistics, Version 24.0.0.0 (IBM Corp. Armonk, NY, USA). General characteristics are presented by mean and standard deviation (SD), median and inter-quartile range (IQR) and significance levels of 5%. With regards to the statistical measures of the NASA-TLX values, literature is inconsistent. Both mean/SD as well as median/IQR reporting can be found. To allow for better comparison with previous data, we report the NASA-TLX in both manners.

To investigate for influencing factors on workload, a multiple logistic regression model with a stepwise selection of the predictor variables was created. The NASA-TLX score was used as the dependent variable while mission characteristics etc. were used as the independent variables.

## Results

### Descriptive statistics

**Fig. 1:**
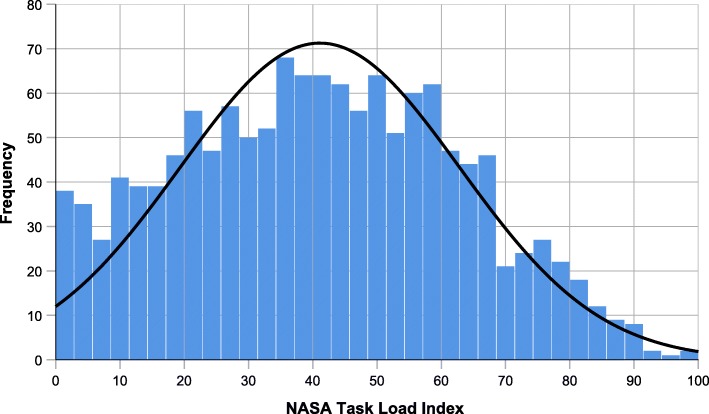
Single-mission global TLX-Scores are frequently reported around medium values of workload. However, a relevant number of missions results in both very high (3.8% >80) and very low (19.3% <20) workload scores

A total of 2048 EMS missions were documented during the course of the study, of which 1744 were classified as emergency missions. Ultimately, 1361 missions, documented by 543 paramedics, fulfilled all quality criteria and were included in our statistical analysis. The values of TLX-scores are subject to large variations. This broad distribution means, that 263 (19.3%) missions were scored with a very low workload of less than 20/100, while 52 (3.8%) missions achieved very high ratings of more than 80/100. The distribution of global NASA-TLX scores is demonstrated in Fig. 1. The mean global mission workload was scored at 41.11/100 (median: 41.00; IQR: 24.25–57.50). Table 3 shows global TLX-scores in comparison to the six TLX subscales.

**Table 3:** Descriptive statistics of TLX and subdimensions

Subdimension	Mean (SD)
Mental demand	52.63 (31.51)
Physical demand	44.62 (32.01)
Temporal demand	44.65 (32.97)
Performance	24.14 (23.42)
Effort	44.64 (29.94)
Frustration	35.76 (30.50)
Global TLX	41.11 (21.77)

**Fig. 2:**
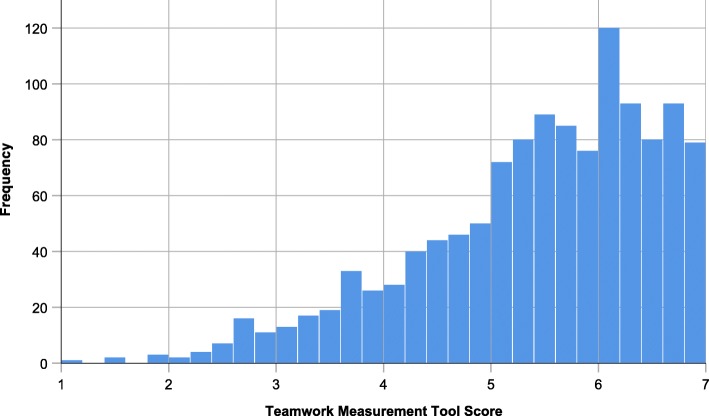
Single-mission teamwork as scored by the teamwork measurement tool is frequently perceived as very positive on the 7-step Likert-scale

Teamwork was rated as very high, both globally (M = 5.56; SD = 1.15) and in all subdimensions. Leadership and Team Coordination gained highest ratings (M = 5.88; SD = 1.15), with Mutual Performance Monitoring (M = 5.52; SD = 1.46) and Verbalising Situational Information (M = 5.19; SD = 1.47) trailing closely. Only a minority of missions was rated with a TMT score below 3.00 (*N* = 40). Figure 2 shows the distribution of global TMT scores with its vast majority of positive ratings.

Table 4 shows the descriptive statistics of the variables used in the multiple linear regression models as mentioned below:

**Table 4:** Descriptive statistics of included candidate variables

	N	% of total missions
Verbally aggressive patient	57	4.20%
Resuscitation	136	10.00%
Polytrauma patient	128	9.40%
Intravenous access	459	33.70%
Intraosseous access	24	1.80%
Adminst. Medication	195	14.30%
Infectious patient	32	2.40%
Missing equipment	43	3.20%
Airway management	111	8,20%

### Multiple linear regression model

With the help of a multiple linear regression model, we could identify predictor variables that are associated with an alteration of the NASA Task Load Index. Of the various candidate variables, only a limited number was identified as relevant factors to affect TLX-scores. Variables the expert panel thought of as being clinically relevant, but had been excluded during the process of stepwise integration into the regression model or had to be excluded due to not being significant are shown in Table 5.

**Table 5:** Variables excluded during the stepwise multiple regression analysis

Diagnoses	Aggression events
Sepsis	Obstruction of treatment
Myocardial Infarction	Physically aggressive bystander
Stroke	Verbally aggressive bystander
Traumatic brain injury	

A one-point increase of the NACA score was associated with an increase of the TLX by 2.71 points (*p* < 0.001). Polytraumatized patients caused an increase of 11.41 points (p < 0.001), while resuscitation scenarios caused an increase of 9.06 points (p < 0.001).

Should the need arise for non-physician rescue workers to establish intravenous access; this would be followed by an 8.20 point increment on the TLX (*p* < 0.001) whereas the need for intraosseous access led to a 7.51 point increment. (*p* < 0.05). The act of administering any medication other than balanced crystalloids was also accompanied by a 3.35 point increase (*p* < 0.05).

**Fig. 3:**
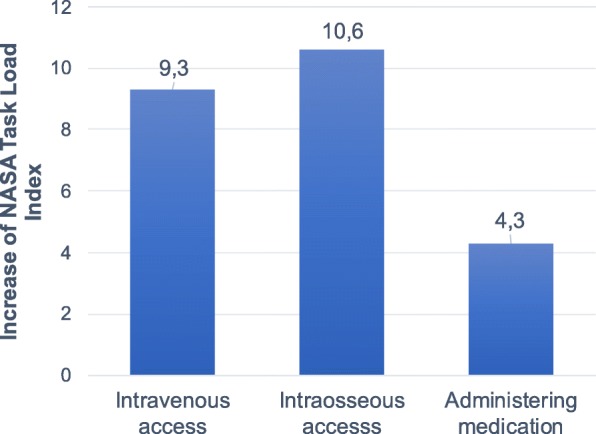
The task load index increases significantly for each mission if paramedics need to perform certain (e.g. invasive) medical procedures

Figure 3 gives a comprehensive overview of the effect of such medical procedures on TLX workload.

Verbally aggressive patients increased the TLX by 8.90 points (*p* < 0.01), while a physically aggressive patient would increase the TLX by 10.54 points (p < 0.01). Being accused by patients or relatives of having made a professional mistake led to an upsurge of the TLX by 14.5 points (*p* < 0.001).

Some additional circumstances were identified by the multiple linear regression models that resulted in an increase of the measured taskload: Transporting patients with transmissible pathogens (delta = 7.94 points; *p* < 0.05), missing necessary equipment (delta = 10.52 points; p < 0.001), and missions causing overtime (delta = 4.88 points; p < 0.05). Every additional kilogram of the patient’s bodyweight caused an increase of the taskload by 0.05 points (p < 0.05).

Good Teamwork was associated with a decrease of the participants’ task load by − 2.18 points per additional point on the 7-step TMT Scale (p < 0.001).

The subjectively perceived indication of a mission was measured on a 5-step Likert-scale (Min = 1 - not indicated; Max = 5 - absolutely indicated; Mean = 4.09; SD 1.35). Every additional step on this scale led to an increase of the TLX by 2.44 points (*p* < 0.01).

The final model’s R^2^ value (coefficient of determination) was calculated at 0.44, so 44% of the variation in taskload can be explained by the model containing the factors mentioned above.

For this statistical model, the scatterplots of standardized predicted values versus standardized residuals showed that the data met the assumptions of homogeneity of variance and linearity and the residuals were approximately normally distributed. Table 6 summarizes the results of the multiple linear regression analysis.

**Table 6:** Results of the stepwise multiple linear regression model

	Unstandardized Coefficients		Standardized Coefficients			95,0% Confidence Interval for B	
	B	Std. Error	Beta	t	Sig.	Lower Bound	Upper Bound
Constant	− 79.03	8.04		−9.83	.000	−94.80	−63.26
NACA Score	2.71	0.45	0.21	6.00	.000	1.83	3.60
Intravenous access	8.20	1.18	0.18	6.93	.000	5.88	10.53
Verbally aggressive patient	8.90	2.68	0.09	3.32	.001	3.65	14.15
Polytrauma	11.41	1.85	0.15	6.16	.000	7.78	15.05
Resuscitation	9.06	2.27	0.13	3.99	.000	4.60	13.52
Being accused of having made a mistake	14.51	3.00	0.11	4.84	.000	8.63	20.38
TMT Score	−2.18	0.43	−0.12	−5.13	.000	−3.01	−1.35
Subjectively felt indication	2.44	0.45	0.15	5.48	.000	1.57	3.32
Missing equipment	10.52	2.60	0.09	4.05	.000	5.43	15.62
Administration of medication	3.35	1.53	0.06	2.19	.029	0.35	6.36
Mission caused overtime	4.88	1.76	0.06	2.78	.006	1.44	8.32
Physically aggressive patient	10.54	4.03	0.07	2.62	.009	2.64	18.44
Infectious patient	7.94	3.17	0.06	2.50	.012	1.72	14.17
Airway management	4.21	2.27	0.06	1.85	.064	−0.25	8.66
Patient’s body weight	0.05	0.02	0.05	2.41	.016	0.01	0.09
Intraosseous access	7.51	3.76	0.05	2.00	.046	0.13	14.89

## Discussion

The ergonomics and human factors of health care providers can have a significant impact on patient outcome in all aspects of medicine. In this, workload has been identified as a key factor to influence human performance. However, data focusing on workload and ergonomics in prehospital emergency medicine is scarce. To amend this, our cross-sectional, observational study provides broad data on perceived workload and an analysis of influencing factors for a large nationwide cohort of paramedics and missions.

When compared to existing data on NASA-TLX scores from other professions and tasks, the reported TLX values from our study (Mean: 41.11, SD: 21.77, Median 41.00 IQR: 24.25–57.50) are lower and show a greater variance. In the largest meta-analysis to date, Grier reports a mean global TLX score of 45.29 (SD: 14.99) across all professions and reports higher TLX scores for medical tasks at median 50.60 (IQR: 39.35–61.45). High-reliability professions such as air traffic control or aircraft piloting also score higher median values at 52.44 (IQR 42.81–68.32) and 47.78 (IQR: 37.70–54.80) respectively [10].

Our analysis of TLX subdimensions allows for a closer look at the separate contributors to workload (Table 3). First and foremost, paramedics perceive their tasks as being mentally demanding. Physical, temporal demands and the need to put in hard effort score almost ten points lower. Encouragingly, the very low ratings on the performance and frustration scales point towards frequent feelings of success and encouragement. These findings are in concordance with data from our recent study on job satisfaction amongst German paramedics. The attitude towards the content of work was found to be very positive, while other aspects of the job such as pay and higher management scored significantly lower [11].

Interestingly, the data point to a maldistribution of global workload levels. Considering the inverse-U-shaped correlation between workload and human performance, it must be postulated that paramedics regularly operate outside their ergonomically optimal range of workload. Especially task-overload will lead to significant decreases in performance output. A substantial number of missions was rated with a very high workload score (3.8% of missions with TLX above 80). Unfortunately, literature does not provide clear cut-off values of the NASA-TLX that would be required to definitely discern those non-ergonomic missions in detail [7, 10]. Nonetheless, science needs to identify and eventually fight those factors pushing workload out of its optimal (i.e. medium) range.

From our data, we are able to identify certain mission characteristics that cause relevant increases in workload. The patients’ medical condition as represented by NACA scoring and tracer diagnoses leads to major increases in TLX-scores as demonstrated in Table 6. Our findings are in concordance with a recent study on a cohort of emergency physicians, who also experienced an increase in perceived workload with increases in patients’ NACA scores [6].

Regarding invasive procedures and the administration of medications German law usually reserves these actions for registered physicians only. Under distinct circumstances, they may be executed legally by paramedics. In our dataset, cases of intravenous or intraosseous cannulation, airway management and administration of any medication other than crystalloids could be identified as independent factors to increase workload in our multivariate analysis, irrespective of the aforementioned severity of the medical condition.

So far, the factors we discussed can be classified as immediately related to the medical nature of the emergency itself. Therefore, they cannot be evaded entirely but enhancements in the professional training of skills and competencies to handle the severely ill or injured may help to mitigate the effect such scenarios have on workload.

Other factors that increase workload are more easily modifiable. Missions during which necessary equipment is found missing or missions in overtime score distinctively higher and could possibly be avoided by improvements in the superordinate local structures of EMS providers. Furthermore, technical advances in loading and transport equipment should be implemented for their possible benefits on workload increments in missions with obese patients (steady increment per kilogram of weight). Education in transmission pathways and training in procedural judgment should be tested to lessen workload of missions involving transmittable pathogens.

Insults and aggression from patients and bystanders were reported in a minority of cases only but had a major impact on workload. This finding is the most disconcerting of all, as such motions are diametrical to the benevolence of medicine in general and the personal efforts and sacrifices of EMS professionals in particular. Sadly, however, these findings are in keeping with recent literature all indicating a steady rise in attacks on medical personnel inside and outside of the hospital [12–16].

On a more positive side, our data also suggests a positive effect of good teamwork on workload (delta TLX − 2.18 points per 1 point increase in TMT score). The net decrease in workload seems small at a first glance, but it should be kept in mind that teamwork scores were very high and with little variance, suggesting a ceiling effect in the relationship of teamwork and workload. In other terms: Teamwork was so consistently good that effects on workload are seemingly smaller than the importance of the relationship might deserve [17].

Our findings on workload and modifiable influencing factors call for immediate efforts to improve ergonomics. In order to optimize workload, negative determinants should be antagonized and levels of workload should be homogenized towards adequate ranges. True to the principles of human factors, emergency medical services should strive to improve system performance while at the same time increase paramedics’ well-being.

This seems of special importance in the lights of a previous study, in which we saw a significantly increased rate of post-traumatic stress disorder and lower levels of well-being amongst German paramedics compared to the general population [18]. While those findings certainly need to be considered as the end-point of a complex multi-factorial process, it seems worthwhile to investigate whether amendments in those factors that increase workload could bring about relevant improvements in paramedics’ job satisfaction, well-being and mental health.

Apart from these insights on workload, our data also provides novel information on the actual frequency with which invasive procedures are performed in German EMS. As demonstrated in Table 4, intravenous cannulation is a common occurrence of about one in three missions, whereas intraosseous access is a rare procedure with a found prevalence of less than 2% of missions. In consequence, the overall mathematical contribution of the factor “intraosseous access” to our regression analysis is minor, with an identical coefficient of determination R^2^ = 0.44 for a hypothetical linear model that would not include this infrequent factor. For additional information on this alternative model, please see the additional online content, Additional file 1.

### Limitations

Naturally, there are some limitations to our study. In spite of our extensive efforts to disseminate the study as widely as possible throughout Germany, there may be an undetectable selection bias regarding those willing to participate. It could very well be that job motivation, performance levels and workload perception of our volunteers are not wholly representative of the population of paramedics in Germany.

Furthermore, the so-called healthy-worker effect may lead to an ongoing selection of only those paramedics staying on the force that are actually capable to withstand the demands of the job and will feel less loaded with work [19].

Thirdly, our investigation centred on the self-reporting of perceived workload. This technique may be influenced by hindsight errors. Temporal distance to the missions may alter recollection and workload scoring. It was due to the countrywide set-up of our study that we could not provide complementary physiological measurements of workload-related parameters or observational assessment techniques.

As a fourth, the subjective perception of workload may always be influenced not only by factors attributable to the mission, but by factors attributable to the person who is performing the task. These latter factors lie outside the scope of this study, but should be investigated in their entire depth in the future.

Summarizing these limitations in numbers, our calculated model accounts for roughly half of the variation of workload. To identify the factors responsible for the other half of variation poses an exciting challenge for future studies to come.

## Conclusion

In our study, we documented medium mean levels of workload in prehospital emergencies. However, relevant numbers of missions generate such high workload that optimal performance may be endangered. We identified several factors related to relevant increases in workload. Interventions to modify these risk-factors should be investigated not only for their effect on workload but also on job satisfaction and on well-being of EMS-personnel.

## Additional file

Additional file 1: Alternate model 1. (DOCX 14 kb)

## Data Availability

The dataset generated and analysed during the current study is available at the Zenodo data repository: 10.5281/zenodo.2532609

## Abbreviations

EMS: Emergency Medical Service
NACA: National Advisory Committee for Aeronautics
NASA: National Aeronautics and Space Administration
TLX: Task Load Index
TMT: Teamwork Measurement Tool
