# Supplementary material for: Single mission workload and influencing factors in German prehospital emergency medicine - a nationwide prospective survey of 1361emergency missions
Source: Scand J Trauma Resusc Emerg Med. 2019 Aug 16;27:75. doi: 10.1186/s13049-019-0650-2 (PMC6698029; doi:10.1186/s13049-019-0650-2)
Supplement: Supplementary file 1 — Alternate model 1. (DOCX 14 kb) [file 13049_2019_650_MOESM1_ESM.docx]

|  | | |  |  |  |  |  |
| --- | --- | --- | --- | --- | --- | --- | --- |
| Results of the stepwise multiple linear regression model  (**without intraosseous access) -**  **coefficient of determination: R^2^ = 0.44** | | |  |  |  |  |  |
|  | **Unstandardized Coefficients** |  | **Standardized Coefficients** |  |  | **95,0% Confidence Interval for B** | |
|  | **B** | **Std. Error** | **Beta** | **t** | **Sig.** | Lower Bound | Upper Bound |
| Constant | -73,564 | 7,567 |  | -9,721 | 0,000 | -88,411 | -58,717 |
| NACA Score | 2,698 | 0,453 | 0,204 | 5,961 | 0,000 | 1,81 | 3,586 |
| Intravenous access | 7,863 | 1,173 | 0,177 | 6,701 | 0,000 | 5,561 | 10,165 |
| Verbally aggressive patient | 8,712 | 2,68 | 0,083 | 3,251 | 0,001 | 3,455 | 13,97 |
| Polytrauma | 11,68 | 1,85 | 0,154 | 6,314 | 0,000 | 8,051 | 15,309 |
| Resuscitation | 9,22 | 2,273 | 0,13 | 4,056 | 0,000 | 4,76 | 13,681 |
| Being accused of having made a mistake | 14,723 | 2,997 | 0,11 | 4,912 | 0,000 | 8,843 | 20,604 |
| TMT Score | -2,168 | 0,425 | -0,115 | -5,096 | 0,000 | -3,002 | -1,333 |
| Subjectively felt indication | 2,475 | 0,446 | 0,155 | 5,55 | 0,000 | 1,6 | 3,35 |
| Missing equipment | 10,59 | 2,601 | 0,09 | 4,072 | 0,000 | 5,487 | 15,692 |
| Administration of medication | 3,831 | 1,514 | 0,065 | 2,53 | 0,012 | 0,86 | 6,802 |
| Mission caused overtime | 4,9 | 1,757 | 0,062 | 2,789 | 0,005 | 1,452 | 8,347 |
| Physically aggressive patient | 10,869 | 4,028 | 0,068 | 2,698 | 0,007 | 2,966 | 18,771 |
| Infectious patient | 8,183 | 3,175 | 0,057 | 2,577 | 0,010 | 1,954 | 14,413 |
| Airway management | 5,2 | 2,218 | 0,069 | 2,345 | 0,019 | 0,849 | 9,551 |
| Patient´s body weight | 0,046 | 0,021 | 0,05 | 2,221 | 0,027 | 0,005 | 0,086 |
